# Supplementary material for: Hyperbaric oxygen protects HT22 cells and PC12 cells from damage caused by oxygen-glucose deprivation/reperfusion via the inhibition of Nrf2/System Xc-/GPX4 axis-mediated ferroptosis
Source: PLoS One. 2022 Nov 10;17(11):e0276083. doi: 10.1371/journal.pone.0276083 (PMC9648730; doi:10.1371/journal.pone.0276083)
Supplement: Attachment — Submitted filename: Response to Reviewers.doc [file pone.0276083.s003.doc]

Journal Requirements:

1. Please ensure that your manuscript meets PLOS ONE's style requirements, including those for file naming. The PLOS ONE style templates can be found at https://journals.plos.org/plosone/s/file?id=wjVg/PLOSOne_formatting_sample_main_body.pdf and 
https://journals.plos.org/plosone/s/file?id=ba62/PLOSOne_formatting_sample_title_authors_affiliations.pdf.

2. We note that the grant information you provided in the ‘Funding Information’ and ‘Financial Disclosure’ sections do not match.

When you resubmit, please ensure that you provide the correct grant numbers for the awards you received for your study in the ‘Funding Information’ section.

3. PLOS requires an ORCID iD for the corresponding author in Editorial Manager on papers submitted after December 6th, 2016. Please ensure that you have an ORCID iD and that it is validated in Editorial Manager. To do this, go to ‘Update my Information’ (in the upper left-hand corner of the main menu), and click on the Fetch/Validate link next to the ORCID field. This will take you to the ORCID site and allow you to create a new iD or authenticate a pre-existing iD in Editorial Manager. Please see the following video for instructions on linking an ORCID iD to your Editorial Manager account: https://www.youtube.com/watch?v=_xcclfuvtxQ.

4. Your ethics statement should only appear in the Methods section of your manuscript. If your ethics statement is written in any section besides the Methods, please move it to the Methods section and delete it from any other section. Please ensure that your ethics statement is included in your manuscript, as the ethics statement entered into the online submission form will not be published alongside your manuscript.

5. Please ensure that you refer to Figure 10 in your text as, if accepted, production will need this reference to link the reader to the figure.

**Author Response:** I have addressed the above additional requirements.

Reviewer #1: The author revealed the neuroprotective effects of hyperbaric oxygen (HBO) in HT22 and PC12 cells, while there are severe problems within the manuscript as listed below:

1. The morphology of PC12 cells is generally round or triangular, and the HT-22 has an elongated/polarized shape. However, the staining results (e.g. Fig 3) demonstrated their PC12 cells did not meet the standard morphology, as we can find that the PC12 here is more of a neuronal-like shape than HT-22. Detail has to be given.

**Author Response:** The PC12 cells in Fig 3 are more numerous, so it appears that the cells has an elongated/polarized shape. In order not to cause misunderstanding, the Fig 3B and Fig 3D have been replaced.

2. The author did not use the standard complete medium (with horse serum) to culture the PC12 cells. Given reasons.

**Author Response:** Sorry for the wrong description due to my carelessness. PC12 cells were maintained in RPMI 1640 medium supplemented with 10% horse serum, 5% fetal bovine serum and 1% penicillin-streptomycin. Modifications have been made in the Materials and methods section.

3. “SLC7ALL” (should be SLC7A11) is everywhere throughout the text, while it became correct in the Discussion and part of the figure (Fig 9), which infers the authors did not get the consensus regarding their research target.

**Author Response:** I'm very sorry for miswriting SLC7A11 as SLC7ALL. They have been modified in the manuscript.

4. The Ferrostatin-1 should serve as an individual group to treat the ferroptosis caused by O-G D/R, rather than being administered together with the HBO. Additional experimental results have to be given.

**Author Response:** A new experiment was added: the cell viability was analyzed when incubated the cells with Ferrostatin-1. As shown in Fig 1C and 1D, like the HBO group, the HT22 and PC12 cell activities of the Ferrostatin-1 groups were significantly higher than those of the model groups. Moreover, PC12 and H9C2 cell activities were higher than those of the HBO groups or the Ferrostatin-1 groups after being treated with Ferrostatin-1 and HBO at the same time. These results indicated that HBO may protect cells from damage by inhibiting ferroptosis.

5. Lack of information: the number of repeats for individual experiments; scale bar for Fig3 and Fig4; abbreviations.

**Author Response:** The missing information has been added in the manuscript.

Reviewer #2: Major comment:

1. Please clarify whether HBOT protects PC 12 cells from damage caused by oxygen-glucose deprivation/reperfusion via the inhibition of ferroptosis or the inhibition of apoptosis and autophagy (previous publication in BIOCELL, 2022). The authors used the same condition of HBOT to treat PC 12 cells. The previous results showed the protection of HBOT for PC 12 cells from damage caused by oxygen–glucose deprivation/reperfusion via the inhibition of cell apoptosis and autophagy. However, in this study, they showed the protection of HBOT for PC 12 cells from damage caused by oxygen–glucose deprivation/reperfusion via the inhibition of cell ferroptosis. Is it possible that apoptosis, autophagy and ferroptosis can exist at the same condition or the same time, since the morphology, biochemical features and regulatory pathway for all are different?

**Author Response:** Our findings and literature reports suggested that apoptosis, autophagy, and ferroptosis may coexist. During ferroptosis, the balance of the intracellular redox system is disrupted, leading to mitochondrial damage, which may promote the occurrence of autophagy, which may act as a feedback loop to further induce ferroptosis [1-3]. Autophagy is like a double-edged sword, which can induce apoptosis and inhibit apoptosis [4-6].

1. Li J, Liu J, Xu Y, Wu R, et al. Tumor heterogeneity in autophagy-dependent ferroptosis[J]. Autophagy,2021, 1-14.

2. Liu J, Yang M, Kang R, et al. Autophagic degradation of the circadian clock regulator promotes

ferroptosis[J]. Autophagy, 2019, 15(11): 2033-2035.

3. Munoz P, Casas J, Megias E, et al. The anti-cancer drug ABTL0812 induces ER stress-mediated cytotoxic autophagy by increasing dihydroceramide levels in cancer cells[J]. Autophagy, 2020, 1-18.

4. Maiuri MC, alckvar E, Kimchi A , Kroemer G. Self-eating and self-killing: crosstalk between autophagy and apoptosis. Nat Rev Mol Cell Biol. 2007 Sep;8(9):741-52.

5. Booth LA, Roberts JL, Dent P. The role of cell signaling in the crosstalk between autophagy and apoptosis in the regulation of tumor cell survival in response to sorafenib and neratinib. Semin Cancer Biol. 2020 Nov;66:129-139.

6. Pang H, Wu T, Peng Z, Tan Q, Peng X, Zhan Z, Song L, Wei B. Baicalin induces apoptosis and autophagy in human osteosarcoma cells by increasing ROS to inhibit PI3K/Akt/mTOR, ERK1/2 and β-catenin signaling pathways. J Bone Oncol. 2022 Feb 1;33:100415.

2. Regarding the pressure and times of HBOT, the authors used 0.25 MPa and 60 min of HBOT to treat the cells. The pressure is higher than that used in clinical treatment. Why and how do the authors choose the proper pressure? If the authors were willing to mimic or simulate the clinical treatment, repetitive treatment of HBOT should be considered. Dose this effect persist after more than one time treatment? Furthermore, the PH value of culture medium may be changed in this condition that could influence the interpretation of protection mechanism.

**Author Response:** Guidelines recommend using a pressure of 2 to 3 times standard atmospheric pressure for HBO therapy. Referring to previous studies, the pressure used for HBO treatment in this study was 2.5 times the atmospheric pressure, which did not exceed the recommended safe range. And, the cell viability was detected by CCK8 assay to determine the time of HBO treatment. All experiments in this study have been repeated three times with consistent results. After the pH test paper detection, the pH value of the culture medium basically did not change under the conditions of this study.

3. Although the authors checked the morphology, related gene and protein expression of ferroptosis for HBOT treated PC cells directly, there was still some difference between HBO group and HBO+Ferrostatin-1 (HBO+F) group. Did that show statistic difference? It seemed that HBO and ferrostatin-1 had synergistic effect in ferroptosis which meant HBOT protection partially via the inhibition of ferroptosis. The authors may try to pretreat model group with ferrostatin-1 before HBOT to see how it goes. Otherwise, the authors need to justify the title based on the current results.

**Author Response:** There was a statistical difference between the HBO group and the HBO+Ferrostatin-1 (HBO+F) group. HBO and ferrostatin-1 have a synergistic effect in inhibiting ferroptosis. A new experiment was added: the cell viability was analyzed when incubated the cells with Ferrostatin-1. As shown in Fig 1C and 1D, like the HBO group, the HT22 and PC12 cell activities of the Ferrostatin-1 groups were significantly higher than those of the model groups. Moreover, PC12 and H9C2 cell activities were higher than those of the HBO groups or the Ferrostatin-1 groups after being treated with Ferrostatin-1 and HBO at the same time. These results indicated that HBO may protect cells from damage by inhibiting ferroptosis.

4. HBOT itself could produce ROS, especially while the higher pressure or the longer duration was used. Although some papers, as the authors cited, indicated HBOT might reduce the oxidative stress in the disease condition, it has been still arguable when HBOT was applied clinically in some neurological disorders, such as stroke. Please describe more to support your view in the discussion section.

**Author Response:** Hyperbaric oxygen (HBO) therapy is a clinical use of pure oxygen in the environment of 2-3 times atmospheric pressure to treat ischemic and hypoxic diseases, such as carbon monoxide poisoning, cerebral infarction, decompression sickness and coronary heart disease, etc., and has obtained certain curative effects. According to the US FDA and the International Society of Underwater Hyperbaric Medicine, 13 clinical indications including decompression sickness, carbon monoxide poisoning, gas embolism, acute craniocerebral injury, and post-radiotherapy tissue damage can be treated with HBO [1-4]. This part is also highlighted in the introduction part.

1. O. Cheng, R.P. Ostrowski, B. Wu, W. Liu, J.H. Zhang, Cyclooxygenase-2 mediates

hyperbaric oxygen preconditioning in the rat model of transient global cerebral

ischemia, Stroke 42 (2) (2011) 484–490.

2. R.P. Ostrowski, G. Graupner, E. Titova, J. Zhang, J. Chiu, N. Dach, D. Corleone, J. Tang, J.H. Zhang, The hyperbaric oxygen preconditioning-induced brain protection is mediated by a reduction of early apoptosis after transient global cerebral ischemia, Neurobiol. Dis. 29 (1) (2008) 1–13.
3. R.E. Rosenthal, R. Silbergleit, P.R. Hof, Y. Haywood, G. Fiskum, Hyperbaric oxygen

reduces neuronal death and improves neurological outcome after canine cardiac

arrest, Stroke 34 (5) (2003) 1311–1316.

4. Z.N. Guo, L. Xu, Q. Hu, N. Matei, P. Yang, L.S. Tong, Y. He, Z. Guo, J. Tang, Y. Yang,

J.H. Zhang, Hyperbaric oxygen preconditioning attenuates hemorrhagic transformation through reactive oxygen species/thioredoxin-interacting protein/nod-like receptor protein 3 pathway in hyperglycemic middle cerebral artery occlusion rats, Crit. Care Med. 44 (6) (2016) e403–e411.

**Comments on Photos and Figures:**

1.Please provide higher resolution images for Figures 3&4.

**Author Response:** Higher resolution images for Figures 3&4 have been provided.

2.Please present the figures differently, such as viability in the curve and intracellular ROS in columns, but combine images and levels of the same cell line with one Fraction.

**Author Response:** The viability at different times was not researched in this study, so it is not suitable to present it as a curve.

While revising your submission, please upload your figure files to the Preflight Analysis and Conversion Engine (PACE) digital diagnostic tool, <https://pacev2.apexcovantage.com/>. PACE helps ensure that figures meet PLOS requirements. To use PACE, you must first register as a user. Registration is free. Then, login and navigate to the UPLOAD tab, where you will find detailed instructions on how to use the tool. If you encounter any issues or have any questions when using PACE, please email PLOS at [figures@plos.org](mailto:figures@plos.org). Please note that Supporting Information files do not need this step.

**Author Response:** Before uploading,PACE have been used to verify the figures.
