# Supplementary material for: Hyperbaric oxygen protects HT22 cells and PC12 cells from damage caused by oxygen-glucose deprivation/reperfusion via the inhibition of Nrf2/System Xc-/GPX4 axis-mediated ferroptosis
Source: PLoS One. 2022 Nov 10;17(11):e0276083. doi: 10.1371/journal.pone.0276083 (PMC9648730; doi:10.1371/journal.pone.0276083)
Supplement: Attachment — Submitted filename: Response to Reviewers.docx [file pone.0276083.s004.docx]

Response to Reviewers

Reviewer #1: The author has addressed most concerns after this round revision, though there are minor problems needed to be corrected:

1. Fig 1. The author has added independent Ferrostatin-1 group as shown in Fig1C&D, so the Fig1A&B demonstrating the same experiment should be deleted.

**Author Response:** Fig1A&B have been deleted.

2. “SLC7ALL” (should be SLC7A11) is still existing in Fig 8B.

**Author Response:** It has been revised in Fig 8B.

Reviewer #2: According to the authors’ responses, some problems were not clarified clearly. Please provide more explanation with revised figures to support the rationale.

1) Although more literature suggests that apoptosis, autophagy, and ferroptosis may coexist under certain conditions, please list the percentage of cells undergoing ferroptosis and apoptosis or autophagy in the model (oxygen-glucose deprivation/reperfusion) group. How did you define the apoptotic body in Fig 6H? Was it under the same microscopic field where ferroptosis occurred? What proportion of apoptotic cells was found?

**Author Response:** Autophagy, apoptosis and ferroptosis exist in the process of cerebral ischemia-reperfusion injury. The formation of apoptotic bodies is because mitochondria, endoplasmic reticulum and other organelles in apoptotic cells are encapsulated by endoplasmic reticulum membrane together with other cytoplasmic components. After autophagosomes fuse with apoptotic cell membranes, autophagosomes are excreted out of cells to become apoptotic bodies. Our preliminary experimental results found that apoptosis and autophagy mainly occurred in oxygen-glucose deprivation for 0.5 h and reperfusion for 24h, and ferroptosis mainly occurred in oxygen-glucose deprivation for 2 h and reperfusion for 24h. This study focused on ferroptosis and did not observe the percentages of apoptosis and autophagy.

2) As the second comments on photos and figures, please present the figures separately, such as viability in the curve and intracellular ROS in columns, but combine images and levels of the same cell line with one fraction. The reason is that the previous research protocol demonstrated the HBOT protective effect against apoptosis and autophagy by using 0.25 MPa for 90 min/day, however, in the current study against ferroptosis HBOT with 0.25 MPa for 60 min/day was used. Were there the different results in cell viability and ROS levels? How did the authors design the two protocols?

**Author Response:** Literatures show that there is a time window for hyperbaric oxygen therapy, from 60-120min. In the pre-experiment for the current study, HBO with 0.25 MPa for 60, 90 min/day was used separately. It was found that the cell viability and ROS levels of 60 and 90 min/day were the same, so HBO with 0.25 MPa for 60 min/day was selected for this study.

3) In the discussion, please explain the potential differential signaling pathways triggering apoptosis or/and ferroptosis in the model (oxygen-glucose deprivation/reperfusion) group. Because some of listed references were not so relevant, please consider a rearrangement and provide more original discoveries on the coexistence of ferroptosis and apoptotic bodies under the same condition. It may further benefit and lighten the application of HBOT in neurological disorders.

**Author Response:** Combined with our previous and current findings, we found that ferroptosis in the oxygen-glucose deprivation/reperfusion group was accompanied by apoptosis. Potential differential signaling pathways triggering apoptosis and ferroptosis in the model (oxy-glucose deprivation/reperfusion) group have been explained in the discussion.

4) The resolution for Figures 3 and 4 remained the same. Please try to treat cells at a proper density (2.5 x105 cells/well) so that you may get a better picture. Some protocols recommend a short-term serum-free DMEM before ROS detection.

**Author Response:** We tried to get the better pictures for Figures 3 and 4. Figure 3 with higher resolution has been re-uploaded. The re-uploaded Figure 4 is considered higher resolution than the first upload.
